# Targeting neoplastic B cells and harnessing microenvironment: the “double face” of ibrutinib and idelalisib

**DOI:** 10.1186/s13045-015-0157-x

**Published:** 2015-05-29

**Authors:** Rossana Maffei, Stefania Fiorcari, Silvia Martinelli, Leonardo Potenza, Mario Luppi, Roberto Marasca

**Affiliations:** Hematology Unit, Department of Medical and Surgical Sciences, University of Modena and Reggio Emilia, Via Del Pozzo 71, 41124 Modena, Italy

**Keywords:** Ibrutinib, Idelalisib, B cell lymphoproliferative disease, Clinical efficacy, Microenvironment, Immune cells

## Abstract

Tyrosine kinase inhibitors (TKIs) targeting signaling molecules downstream B cell receptor (BCR) are powerfully spreading in the therapeutic landscape of B cell lymphoproliferative disease, due to a manageable toxicity profile and encouraging clinical effectiveness. In particular, ibrutinib, previously called PCI-32765, is a potent inhibitor of Bruton tyrosine kinase (Btk), recently approved for the treatment of relapsed mantle cell lymphoma (MCL) and chronic lymphocytic leukemia (CLL). Moreover, idelalisib (formerly GS-1101 and CAL-101) is a selective reversible inhibitor of the p110δ isoform of phosphoinositol 3 kinase (PI3K) approved for the treatment of patients with relapsed follicular lymphoma (FL) and CLL. These agents directly affect the neoplastic clone, disrupting the supportive platform provided by BCR signaling cascade and by other microenvironmental mutualistic interactions, and also interfering with chemokine gradients and adhesive properties of neoplastic B cells. In the present review, we describe the clinical efficacy of ibrutinib and idelalisib in CLL and B cell non-Hodgkin lymphoma (B-NHL), then focusing on the mode of action (MOA) of these TKIs towards the neoplastic B cell compartment. At last, the review would further expand the view on potential additional targets of ibrutinib and idelalisib belonging to other microenvironmental cellular elements.

## Introduction

Signaling from the B cell receptor (BCR) is essential for normal B cell development and controls several cellular functions such as proliferation, apoptosis, differentiation, and cell migration [1]. Constitutive activation of BCR is a common hallmark of B cell malignancies, including chronic lymphocytic leukemia (CLL), mantle cell lymphoma (MCL), follicular lymphoma (FL), and diffuse large B cell lymphoma (DLBCL), observation that has led the design of a novel panel of inhibitors targeting kinases responsible for BCR signal transduction. In particular, Bruton tyrosine kinase (Btk) and phosphoinositol 3 kinase (PI3K) δ are pivotal signaling molecules early positioned downstream the BCR cascade. Ibrutinib, previously called PCI-32765, is a potent (IC_50_ 0.5 nmol/L) inhibitor of Btk that is inactivated through irreversible covalent binding to Cys-481 in the ATP-binding domain of the kinase. Idelalisib (formerly GS-1101 and CAL-101) is a selective reversible inhibitor of the p110δ isoform of PI3K. The encouraging results of clinical trials using these tyrosine kinase inhibitors (TKIs) envision a possible shift towards a non-chemotherapy treatment era in B cell lymphoproliferative diseases.

In the present review, we focused on ibrutinib, approved by the Food and Drug Administration (FDA) in November 2013 for the treatment of relapsed MCL and in February 2014 as a second-line treatment for CLL and idelalisib, which was approved by FDA in July 2014 for the treatment of patients with relapsed FL or relapsed small lymphocytic lymphoma (SLL) and CLL, pointing the attention to the clinical efficacy in CLL and B cell non-Hodgkin lymphomas (NHL), the antitumor mode of action (MOA) and the possible effects of these drugs on non-malignant cells in tumor microenvironment.

## Clinical efficacy of TKIs

### Ibrutinib

Since the first evaluation of ibrutinib in a phase 1 trial in a wide setting of B cell malignancies, it was demonstrated that this orally administered molecule was well tolerated and induced clinical objective responses (60 %) across B cell histologies, with highest response rate in MCL and CLL patients [2]. Ibrutinib treatment was then investigated in relapsed CLL patients in a phase 1b/2 multicenter clinical trial conducted on 85 patients (33 % with 17p13 deletion), reporting an overall response rate (ORR) of 71 %, with two complete responses (CR), and further 18 % with partial response (PR) with lymphocytosis. At 26 months, the progression-free survival (PFS) was 75 % [3]. The response to ibrutinib did not vary according to traditional high-risk prognostic factors, with 68 % of response in 17p13-deleted setting, even if most events associated with disease progression occurred in patients with high-risk cytogenetics (PFS = 57 %) (Table 1). The most common adverse events were diarrhea, fatigue and upper respiratory tract infections. The events of infection mainly occurred in the early phase of treatment, but collectively, the incidence of grade 3 infections did not result increased as compared to rates reported after traditional therapies. It was also reported that the levels of IgG and IgM remained relatively stable, whereas IgA increased throughout treatment [3]. The efficacy of ibrutinib in previously treated CLL was also confirmed in a phase 3 multicenter study in which 391 patients were randomized to receive ibrutinib or anti-CD20 antibody ofatumumab [4]. The ORR was 43 % (all partial responses) with additional 20 % PR with lymphocytosis in ibrutinib-treated patients as compared to only 4 % in the ofatumumab arm. Ibrutinib in comparison to ofatumumab significantly prolonged the duration of PFS and the rate of OS, regardless of high-risk features. The frequencies of any grade or grade >3 infections in the ibrutinib group were 70 and 24 %, respectively [4]. Very recently, Byrd and colleagues reported updated observations indicating that ibrutinib treatment is well tolerated in CLL patients for an extended period (3-year follow-up) and responses are durable and improves in quality (median time to CR, 21 months), being disease progression uncommon, and primarily occurring in the high-risk patient settings with 17p or 11q deletions in leukemic clone [5]. Ibrutinib is currently investigated in combination with both chemotherapy and monoclonal antibodies to reduce the extent of lymphocytosis and to achieve higher frequencies of complete responses. Burger and colleagues reported the results of a phase 2 study conducted on 40 high-risk CLL patients treated with ibrutinib plus rituximab [6]. The ORR was 95 %, with 87 % PR and 8 % CR, and the PFS at 18 months was 78 % for all patients and 72 % for 17p-deleted/mutated patients. Infections were the most common adverse effects. Overall, the combination strategy was associated with a shorter duration of lymphocytes redistribution as compared to ibrutinib alone, and complete remissions were achieved in more patients. A randomized study comparing ibrutinib versus ibrutinib plus rituximab is ongoing (NCT02007044). As first-line monotherapy, a phase 1b/2 multicenter trial conducted on 29 elderly symptomatic CLL patients reported encouraging results with 71 % ORR (13 % CR) and rare hematological toxicity and infections, indicating that ibrutinib treatment is well tolerated and effective in elderly setting. Median serum levels of IgA increased during treatment, no changes in IgM and IgG over time [7]. As upfront strategy, two phase 3 trials are ongoing comparing the efficacy of chlorambucil or ibrutinib in elderly CLL (RESONATE-2, NCT01722487) and the efficacy of bendamustine-rituximab versus ibrutinib-rituximab versus ibrutinib (NCT01886872). In a very recent phase 1b study evaluating the safety and efficacy of ibrutinib in combination with chemoimmunotherapy (CIT) (bendamustine, rituximab (BR); fludarabine, cyclophosphamide and rituximab (FCR)) in patients with relapsed/refractory CLL, the ORR in the BR-ibrutinib group was 93 % with 40 % CR with an expected toxicity profile and PFS 70 % at 36 months; all three patients receiving FCR-ibrutinib achieved CR. The treatment-related lymphocytosis was reduced, but not completely absent when ibrutinib was combined to BR [8]. A phase 3 randomized trial combining ibrutinib with BR in relapsed/refractory CLL/SLL (NCT01611090) and a phase 2 study combining ibrutinib with FCR in untreated, young CLL patients (NCT02251548) are ongoing. Another recent report of a phase 2 study conducted on 51 treatment naïve and relapsed/refractory CLL patient harboring TP53 aberrations reported activity of single-agent ibrutinib in this high-risk subset with ORR at 24 weeks of 92 % (50 % PR and 42 % PR with lymphocytosis) and PFS at 24 months of 82 % [9]. Rapid disease control was achieved in all tissue compartments and durable responses were reported, but deep remissions were rare, even in previously untreated patients. Noteworthy, subclones carrying 17p13 deletion seemed equally sensitive to ibrutinib as those without the deletion [9].

**Table 1:** Clinical trials with ibrutinib and idelalisib in CLL patients

Study	First line or treated subset	Phase	Number of patients	Age, median (range)	Scheme	ORR (CR)	PFS	TP53 (%)	ORR (CR)	PFS
						All cases	All cases		TP53 subset	TP53 subset
Byrd, NEJM 2013 [3]	Relapsed	1b/2	85	66 (37–82)	Ibru mono	71 + 18^a^ (2 %)	75 % at 26 ms	33 %	68 % (4 %)	57 % at 26 ms
O’Brian, Lancet Oncol 2014 [7]	First line	1b/2	29	71 (65–84)	Ibru mono	71 + 13 %^a^ (13 %)	96 % at 24 ms	6 %	NA	NA
Farooqui, lancet Oncol 2015 [9]	Treated or untreated with TP53 aberrations	2	51	62 (33–82)	Ibru mono	-	-	100 %	50 + 42 %^a^	82 % at 24 ms
Byrd, NEJM 2014 [4]	Relapsed/refractory	3	391	67 (30–86)^b^	Ibru vs. Ofa	43 + 20 %^a^ (0 %)	88 % at 6 ms	32 %	NA	83 % at 6 ms
Burger, Lancet Oncol 2014 [6]	High-risk previously treated or untreated	2	40	63 (35–82)	Ibru + RTX	95 % (8 %)	78 % at 18 ms	50 %	100 % (10 %)	72 % at 18 ms
Brown, Blood 2014 [14]	Relapsed/refractory	1	54	63 (37–82)	Ide mono	39 + 33 %^a^ (0 %)	50 % at 16 ms	24 %	54 % (0 %)	50 % at 3 ms
Furman, NEJM 2014 [15]	Relapsed	3	220	71 (48–90)^b^	Ide + RTX vs. placebo	81 % (0 %)	93 % at 6 ms	38 %	NA	NA

*Ibru* ibrutinib, *Ide* idelalisib, *RTX* rituximab, *Ofa* ofatumumab, *mono* monotherapy, *ORR* overall response rate, *CR* complete response, *PFS* progression-free survival; *ms* months, *NA* not available

^a^The percentages are the ORR (CR and PR) + the PR with persistent lymphocytosis

^b^Data of ibrutinib or idelalisib arm

Ibrutinib also showed antitumor activity in several types of NHL as single agent or in combination [2, 10]. Wang et al. reported the results of a phase 2 study conducted on 111 patients with relapsed or refractory MCL treated with a daily dose of 560 mg of single-agent ibrutinib. The treatment showed durable efficacy with ORR of 68 % (21 % CR) and PFS of 14 months [11]. There was an increase of MCL cells in blood 10 days after treatment initiation in 34 % of patients, with a subsequent decline in these cells to near baseline by day 28 [11]. In patients with relapsed DLBCL, ibrutinib showed preferential activity against tumors with the activated B cell-like (ABC) subtype with a response of 40 % [12]. In a phase 1b study, 32 patients with B-NHL received ibrutinib plus rituximab, cyclophosphamide, doxorubicin, vincristine, and prednisone (R-CHOP), showing promising results, also in the subset of DLBCL, and acceptable safety profile with known toxicities associated with R-CHOP treatment [13]. A phase 3 clinical trial (NCT01855750) to assess the clinical outcome of ibrutinib plus R-CHOP in patients with ABC-DLBCL lymphoma is ongoing.

### Idelalisib

Idelalisib was first evaluated in a phase 1 trial conducted on 54 relapsed/refractory CLL patients, showing an ORR of 72 % with 39 % PR and 33 % PR with treatment-induced lymphocytosis and median PFS of 16 months for all patients. In 13 patients harboring 17p13 deletion and/or TP53 mutation, the ORR was 54 % and median PFS of 3 months (Table 1). In addition, idelalisib was well tolerated, not leading to myelosuppression or an increase in risk of infection as compared to the level already reported in the heavily pretreated CLL population [14]. The combination of idelalisib plus rituximab was inspected in 220 relapsed CLL in a phase 3 multicenter randomized trial that reported acceptable safety profile and improvement in ORR (81 vs. 13 %, all PR), in PFS at 6 months (93 vs. 46 %) and in OS at 12 months (92 vs. 80 %) in the idelalisib group as compared to the placebo group [15]. As in the case of ibrutinib, the addition of rituximab to idelalisib blunted and shortened the duration of treatment-related lymphocytosis.

Idelalisib was also evaluated in two phase 1 studies [16, 17], enrolling 40 patients with relapsed/refractory MCL and 64 patients with relapsed indolent NHL, respectively. In MCL, the ORR was 40 % with 85 % of patients having a reduction in lymph node size and 5 % of CR. Treatment-related lymphocytosis was infrequent in MCL setting and the median PFS was 3.7 months [16]. The response rates reported in MCL treated with idelalisib are comparable to those obtained with other single-agent treatments, including bortezomib, lenalidomide and temsirolimus, but the response duration seems particularly brief. Idelalisib is well tolerated and active also in heavily pretreated, relapsed/refractory patients with indolent NHL, including FL, SLL, marginal zone lymphoma (MZL), and lymphoplasmacytic lymphoma (LPL), showing ORR of 47 % and median PFS at 7.6 months [17]. Gopal et al. reported the results of a phase 2 trial conducted on 125 patients with relapsed indolent NHL treated with single-agent idelalisib confirming the antitumor efficacy (ORR = 57 %, with 6 % CR and median PFS of 11 months) and an acceptable safety profile with low rates of discontinuation due to toxicity and a low incidence of severe adverse events in this setting [18]. Phase 3 trials of idelalisib in combination with rituximab (NCT01838434) and bendamustine/rituximab (NCT01569295) are underway.

## Ibrutinib and idelalisib targeting neoplastic B cells

### Ibrutinib

Uncontrolled BCR signaling plays a major role in the development and progression of B cell NHL and CLL. Btk is required for intracellular transduction of BCR signaling. In fact, Btk is activated by the upstream Src-family kinases Blk, Lyn, and Fyn, and in turn, phosphorylates phospholipase-Cγ (PLCγ) leading to calcium mobilization and activation of nuclear factor κB (NF-κB), and mitogen-activated protein kinase (MAPK) pathways. Moreover, Btk mutations cause the inherited disease X-linked agammaglobulinemia (XLA) in human and X-linked immunodeficiency (Xid) in mice, implying lack of peripheral blood B cells and low levels of serum immunoglobulins [19, 20]. So, the primary deficit associated with Btk disruption is B cell specific. In addition, Btk is involved in signaling of other cell-surface receptors, such as chemokine receptors and adhesion molecules that are essential for B cell trafficking and homing. Btk belongs to tyrosine kinase expressed in hepatocellular carcinoma (Tec) kinase family also including Bmx, inducible T cell kinase (Itk), resting lymphocyte kinase (Rlk) and Tec, preferentially expressed in the hematopoietic system. In particular, Tec/Btk and Itk/Rlk are mediators of BCR and T cell receptor (TCR) signaling, respectively. Itk is also expressed in mast cells and natural killer (NK) cells.

**Fig. 1:**
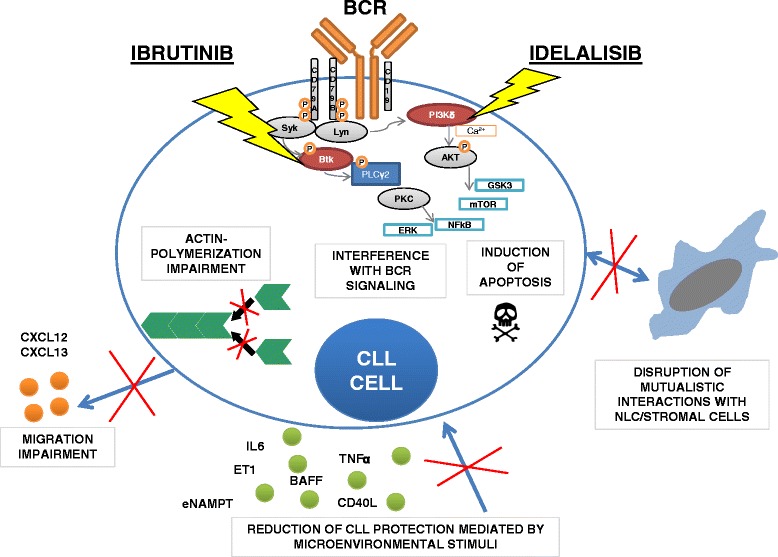
Ibrutinib and idelalisib target CLL cells. The picture summarizes the main mechanisms of action of ibrutinib and idelalisib in CLL. In particular, both agents are able to interfere with BCR signaling cascade by inhibiting the phosphorylation of Btk and PI3Kδ molecules respectively, thus limiting the activation of pivotal pro-survival pathways in CLL cells, i.e., NF-κB, ERK, mTOR, and GSK3 pathways. As consequence, ibrutinib and idelalisib induce pro-apoptotic signaling on CLL cells. They also impair actin polymerization and interfere with chemokine and adhesion receptors, thus leading to ineffective migration and adhesion. Ibrutinib and idelalisib hamper protective stimuli mediated by soluble factors and by interaction with NLC and stromal cells. *BCR* B cell receptor, *Btk* Bruton tyrosine kinase, *PI3Kδ* phosphoinositol 3 kinase δ, *NLC* nurse-like cells

Btk shows constitutive activity in CLL cells and is a critical kinase for CLL development and expansion [21]. Ibrutinib exerts a direct effect by disrupting the BCR and NF-κB signaling. In particular, ibrutinib MOA comprises (i) modest induction of apoptosis dependent on caspase pathway activation, (ii) inhibition of proliferation, (iii) prevention of CLL response to survival stimuli provided by microenvironment, and (iv) impairment of migration and adhesion [22–24] (Fig. 1). Treatment with ibrutinib also mediates apoptotic stimuli on normal B cells but at lower extent in respect to CLL cells. CLL cells have been shown to effectively increase the survival in response to several microenvironmental stimuli such as CD40L, B cell activating factor (BAFF), TNFα, IL6 and IL4 or contact with stromal cells and nurse-like cells (NLCs). Ibrutinib significantly counteracted the protection mediated by such stimuli on CLL cells [23, 22]. Noteworthy, CpG- and NLC-induced CLL proliferation was also inhibited by treating cells with ibrutinib. In NOD/SCID/γchain (NGS) mouse xenograft model, Herman et al. [25] recapitulated ibrutinib-mediated inhibition of BCR and NF-κB signaling, attenuation of CLL proliferation and survival, and reduction of tumor burden in vivo [25]. Concordantly, ibrutinib was shown to down-regulate BCR and NF-κB signaling, decrease the expression of surface activation markers CD69 and CD86, and reduce proliferation of CLL cells from both peripheral blood and tissue compartments in treated patients [26, 27]. In addition, treatment with ibrutinib impaired actin polymerization and migration in response to tissue-homing chemokines (CXCL12, CXCL13) and also down-regulated the expression of BCR-dependent chemokines (CCL3, CCL4). The ability of ibrutinib to cause transient early lymphocytosis and profoundly inhibit CLL progression was modeled in an adoptive transfer TCL1 mouse setting, resembling the transient surge in peripheral lymphocyte count characteristically observed in CLL patients treated with ibrutinib [22]. In fact, in ibrutinib-treated CLL patients, a fraction of leukemic cells equal to 23 % of tissue disease burden redistributes into the blood, leading to a rapid resolution of enlarged lymph nodes [28]. Overall, it was clearly demonstrated that ibrutinib impairs BCR- or chemokine-controlled integrin-mediated adhesion or migration, implying attenuated retention and homing of CLL cells in tissue compartment, thus explaining transient peripheral lymphocytosis seen in treated patients.

Davis et al. [29] firstly identified Btk in a siRNA screen as an essential kinase for survival of ABC-DLBCL, indicating ibrutinib as an agent able to promote apoptosis [29]. In spontaneous canine lymphoma model, ibrutinib was reported to efficaciously inhibit BCR signaling downstream of Btk and induce objective clinical responses [30]. It was clearly demonstrated that BCR and MYD88 signaling pathways together with sustained expression of IRF4 promote ABC-DLBCL survival by inducing NF-κB. Treatment of ABC-DLBCL cells with ibrutinib decreased IRF4 protein, reduced NF-κB signaling, increased interferon (IFN) β production, and also synergized with lenalidomide in killing lymphoma cells in vitro and in mouse model [31]. Furthermore, Btk is robustly expressed in malignant plasma cells from >85 % of multiple myeloma (MM) patients and in lymphoplasmacytic cells from Waldenström macroglobulemia (WM). In MM, ibrutinib was shown to decrease CXCL12-mediated migration, down-modulate CCL3, and affect MM cell growth and survival triggered by IL6 or contact with stromal cells [32, 33]. Similar inhibitory effects on proliferation and survival, CCL3/CCL4 secretion, and CXCL12 signaling occur in hairy cell leukemia (HCL) treated with ibrutinib [34].

### Idelalisib

PI3K pathway is well known as a key survival mechanism in many cancers, including CLL and B-NHL. It integrates and transmits signals from several surface receptors including BCR, chemokine receptors and adhesion molecules, thereby controlling survival, proliferation and migration [35]. PI3K type I enzyme converts PtdIns(3,4)P_2_ into PtdIns(3,4,5)P_3_ that recruits in the cell membrane via binding to pleckstrin homology domain several downstream signaling molecules such as Tec kinases, Akt, integrin-linked kinase, and Rac guanine exchange factor. The δ isoform is one of the four catalytic isoforms of PI3K class I, also comprising p110α, p110β, and p110γ. The p110α and p110β are ubiquitously expressed, whereas the p110δ isoform is generally restricted to hematopoietic cell type and highly expressed in lymphoid cells. B cell defects, comprising lack of B1 lymphocytes, reduced number of mature B cells and impaired antibody production were reported in mice with deleted or mutated PI3Kδ, whereas knockout mice for PI3Kγ showed predominantly T cell defects [36].

PI3Kδ is homogenously expressed in CLL cells and also present in normal B cells, even if it shows higher intrinsic activity in leukemic cells. It is also expressed in normal T cells and NK cells [37, 38]. Pre-clinical studies demonstrated that idelalisib exerts a dose-dependent cytotoxicity on CLL cells primarily by induction of caspase-dependent apoptosis, instead showing less effect on normal B cells. In CLL cells, idelalisib abrogates the protective effect of many microenvironmental signals including CD40L, BAFF, TNFα, ET1, fibronectin adhesion as well as contact with stromal cells and NLC [38–40] (Fig. 1). The PI3Kδ signaling is essential for B cell response after BCR stimulation [41, 42]. Accordingly, when CLL cells were treated with idelalisib, the pro-survival effect of BCR stimulation was abrogated [39]. Clinical observations in idelalisib-treated CLL patients evidenced a redistribution of CLL cells from tissue to peripheral blood, suggesting a possible interference of idelalisib with leukemic cell migration and adhesion. In vitro studies demonstrated that idelalisib inhibits CLL chemotaxis and adhesion to stromal and endothelial layers [39, 43]. Noteworthy, it was demonstrated that idelalisib sensitizes CLL to the cytotoxic action of several drugs, such as fludarabine, bendamustine and dexamethasone, and also prevents some lenalidomide effects on CLL, i.e., cell activation, internalization of CD20 and secretion of pro-angiogenic factors [44, 39]. Moreover, idelalisib dramatically increased apoptosis mediated by histone deacetylase inhibitors in NHL and CLL cells [45]. MCL and MM cells show constitutive activation of Akt that is dependent on PI3Kδ signaling. Thus, idelalisib induced apoptosis on MCL and overcame MM growth conferred by IL6, insulin-like growth factor 1 and co-culture with marrow stromal cells [46, 47].

## Ibrutinib and idelalisib harnessing microenvironmental cells

### NK cells

**Fig. 2:**
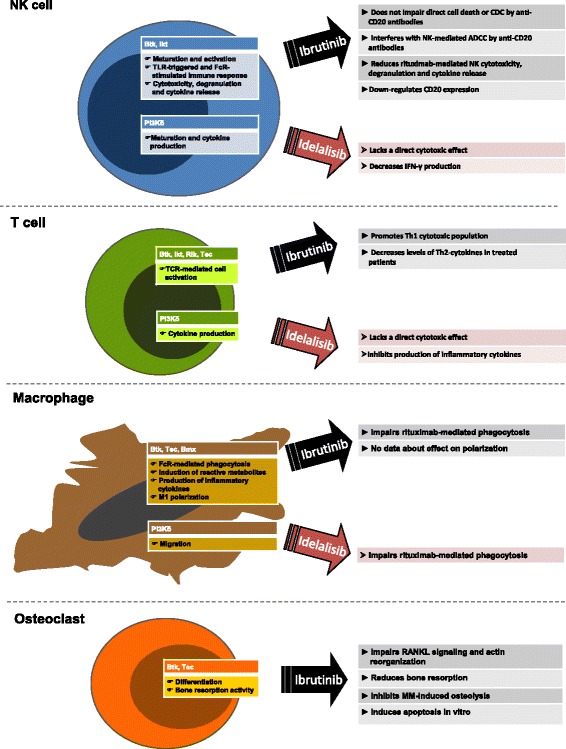
Schematic view of the role of Btk and PI3Kδ molecules in non-malignant cells. The scheme summarizes the main cellular functions, in which Bruton tyrosine kinase (Btk) phosphoinositol 3 kinase (PI3K) δ molecules are involved in NK cells, T cells, macrophages, and osteoclasts. On the right section, some data concerning the effects of ibrutinib and idelalisib on these cells are indicated. *NK* natural killer, *TLR* toll-like receptor, *CDC* complement-dependent cytotoxicity, *ADCC* antibody-dependent cell-mediated cytotoxicity, *IFNγ* interferon γ, *TCR* T cell receptor, *OC* osteoclasts, *MM* multiple myeloma

NK cells are innate effectors that recognize and eliminate virally infected and transformed cells, by secreting preformed granules containing perforin and granzymes and also by inducing apoptosis of target cells throughout activation of death receptors. Btk is not only critical for the B development and differentiation but also regulates NK cell innate function. NK cells up-regulate Btk expression during maturation and activation, and NK cells lacking Btk show reduced toll-like receptor (TLR)-triggered immune response with low expressions of IFNγ, perforin, and granzyme B and impaired cytotoxic activity [48]. Btk has also a critical role in regulating NK activation in response to antigen-presenting cells [49]. In addition, Itk mediates calcium mobilization, granule release and cytotoxicity in Fc receptor (FcR)-stimulated NK [50]. In line with these data, the inhibition of Btk and Itk by ibrutinib might impair NK function and counteract the mechanisms of action of therapeutic anti-CD20 antibodies (rituximab, obinutuzumab and ofatumumab) including FcR stimulation and antibody-dependent cell-mediated cytotoxicity (ADCC). Rituximab is currently approved for the treatment of B cell NHL and CLL in combination with chemotherapy, whereas ofatumumab and obinutuzumab are in clinical trials. All therapeutic anti-CD20 antibodies act throughout immune-mediated mechanisms, including complement-dependent cytotoxicity (CDC), ADCC and antibody-dependent phagocytosis (ADCP) by macrophages and neutrophils, and also through direct cell death in case of obinutuzumab. Ibrutinib was reported to inhibit rituximab-induced NK cell cytokine secretion, NK cytotoxic effect towards coated tumor cells and also to prevent FcR-stimulated NK cell degranulation. Thus, it seems that the concurrent administration of ibrutinib and rituximab determines an antagonistic effect in vitro, with the reduction of antitumor efficacy of rituximab as a result of ibrutinib-mediated inhibition of FcR-stimulated NK cell function, specifically ADCC [51] (Fig. 2). It is likely that the ibrutinib-mediated impairment of NK functions is mainly driven by Itk inhibition. As consequence, selective Btk inhibitors or alternative ibrutinib dosing schedule may overcome the observed antagonistic effect of these agents. Very recently, De Roit et al. demonstrated that pretreatment with ibrutinib does not impair direct cell death or complement-mediated lysis of leukemia cells by anti-CD20 antibodies but strongly inhibits all cell-mediated mechanisms [52].

Idelalisib was reported to inhibit the immune cell-mediated mechanisms of anti-CD20 antibodies even if at weaker extent as compared to ibrutinib [52]. In addition, ibrutinib, fostamatinib and idelalisib were reported to down-regulate CD20 expression in some cell lines and in primary CLL and MCL samples in another study [53]. Noteworthy, the PI3Kδ-specific signaling has a key role in NK maturation and cytokine production [54]. In this contest, idelalisib lacks a direct cytotoxic effect on NK cells but decreases NK production of IFNγ [38]. However, in another study, fosfamatinib and ibrutinib, but not idelalisib, were shown to affect rituximab-induced NK degranulation and cytotoxicity as well as NK secretion of TNF and IFNγ [53]. Of interest, the dual PI3K p110δ and p110γ inhibitor, IPI-145, also affects NK cells by promoting cell death [55].

In this scenario, it has also to be considered that in vivo the egress of neoplastic B cells from tissues to periphery mediated by ibrutinib and other kinase inhibitors may expose cells to a more abundant and fully active complement compartment favoring the CDC induction by concurrent administration of anti-CD20 antibodies, mitigating the potential disadvantage of reduced ADCC. Indeed, encouraging clinical results of ibrutinib in combination with rituximab in high-risk CLL patients in phase 2 study [6], and idelalisib in combination with rituximab in a phase 3 study [15], are reported. However, studies testing different timing in the administration of the anti-CD20 antibodies relative to initiation of ibrutinib or idelalisib or modification of dosing schedules should be carried out to unravel the possible interactions between these drugs at molecular levels and thus maximize the clinical efficacy of the therapeutic combination.

### T cells

Tumor immune surveillance hinges upon the capacity to elicit a robust Th1 and CD8 T cell response that promotes cytotoxic effects with the production of IFNγ and IL2. CLL subversion of immune response comprises the disruption of T cell functions with impairment of immune synapse formation, alteration of actin rearrangement and signal transduction, and reciprocal induction of inhibitory receptors and ligands [56–59]. Moreover, CLL cells secrete a panel of cytokines that promote the aberrant recruitment of a Th2-dominant response, thus interfering with an effective direct T cell cytotoxicity and also favoring the maintenance of a prosurvival niche for leukemic cells through IL4, IL10, and CD40 stimulation.

In T cells, three Tec kinases are expressed, Itk, Rlk/Txk, and Tec. Itk is expressed at highest amounts and plays the major role in regulating signaling from the TCR, starting from phosphorylation of PLCγ that leads a cascade of events comprising the activation of nuclear factor of activated T cells (NFAT), NF-κB, and MAPK pathways as well as the induction of calcium mobilization, reorganization of actin cytoskeleton, synapse formation, and cell adhesion. Ibrutinib is an irreversible inhibitor of Itk, leading to a decrease in PLCγ phosphorylation, a downstream inactivation of IkBα, JunB, and NFAT signaling, and a reduction of intracellular calcium flux in response to TCR stimulation in primary CD4+ cells and in Jurkat T cell line [60]. In Th1 CD4 and CD8 T cells, Rlk plays a redundant role to Itk in response to TCR stimulation. As consequence, ibrutinib at clinical relevant doses is able to influence the Th1/Th2 polarization of CD4+ population by decreasing the Th2-dominant transcription factor JunB, whereas inducing the Th1-specific transcription factor T-bet. It turns out in a prevalence of IFNγ-producing Th1 in respect to IL4-producing Th2 cells. The Th1 induction was also sustained by the observation in CLL patients treated with ibrutinib, showing a decrease level in serum of Th2-type cytokines such as IL4 and IL10 at day 28 of treatment compare to baseline. Overall, the study of Dubovsky and colleagues [60] provides evidence that ibrutinib may influence T cell function, by skewing T cells from a Th2-dominant to a Th1 and CD8+ cytotoxic population, due to a selective Itk inhibition and a compensatory mechanism mediated by Rlk that remains uninhibited in Th1 cells (Fig. 2). Accordingly, mice deficient in Itk showed impairment in Th2 polarization and Th2 cytokine production in response to Th2-inducing agents and infections in vivo [61, 62].

Cytokine production by T cells is mediated by PI3K signaling. Idelalisib did not show any direct cytotoxic effect on T cells, but it could inhibit the production of several inflammatory cytokines such as IL6, IL10, TNFα, and CD40L [38]. Similarly, the inhibition of both PI3K p110δ and p110γ by IPI-145 was reported to reduce the secretion of IL2, TNFα, and IFNγ by TCR-stimulated T cells [55]. Few correlative observations are reported in published clinical trials. Burger et al. showed a reduction of CD3+CD4+ and CD3+CD8+ T cell count in the periphery of patients treated with ibrutinib and rituximab after 6 and 12 months of treatment [6]. In phase 1 trials testing single-agent idelalisib on relapsed/refractory CLL or refractory indolent NHL, a correlative study did not show any significant changes in the absolute number of CD4+/CD3+ T helper cells, CD8+/CD3+ cytotoxic T cells or CD56+/CD16+/CD3− NK cells in peripheral blood of treated patients [14, 17].

### Macrophages

Macrophages are involved in the essential process of engulfment and clearing of microbial pathogens. In particular, the ingestion of IgG-opsonized targets initiates with the engagement of FcγRs, activation of multiple downstream signaling pathways and actin polymerization, thus leading to the formation of phagocytic cup, the extension and fusion of pseudopods, and the internalization and maturation of phagosomes, at which the clearing of pathogens occurs.

Among cytoplasmic tyrosine kinases of Tec family, macrophages express Btk, Tec and Bmx. The involvement of Btk in FcγR-mediated phagocytosis was first reported in monocytes from XLA patients and also in peritoneal macrophages from Xid mice lacking functional Btk [63, 64]. Furthermore, Btk seems irrelevant for macrophage functions associated with T cell activation such as antigen presentation, co-stimulation, or production of T cell-directed cytokines but instead plays a major role in “innate effector” functions such as phagocytosis, induction of reactive metabolites, production of proinflammatory cytokines and microbicidal activity.

Btk and Tec kinases are required for optimal FcγR-mediated phagocytosis. Both kinases are activated throughout phagocytosis in primary macrophages and inhibition of Btk by small interfering RNA markedly reduced Mac-1 activation and FcγR-induced phagocytosis [65]. In particular, the first stage of Tec kinases activation rapidly occurs at nascent phagosomes with their recruitment to the membrane through their pleckstrin homology domain. Btk and Tec are also involved in the transition of integrin from a cytoskeleton-bound resting to a mobile state at phagosome cup. It is also likely a subsequent involvement of Btk and Tec in later stages of phagosome maturation with distinct roles. Thus, Btk function is involved in phagocytosis by sustaining the late and prolonged activation of PLCγ2 [65]. In accordance with these results, recent studies indicated that pharmacological inhibition of Btk by ibrutinib is able to impair the phagocytosis of rituximab-coated CLL cells by macrophages at clinically relevant doses [52, 66] (Fig. 2). Macrophages also expressed the ubiquitous isoforms of PI3K, p110α and p110β as well as the hematopoietic enriched p110δ isoform. PI3K has been demonstrated to regulate phagocytosis and migration of macrophages [67]. The inhibition of PI3Kδ by idelalisib was reported to reduce rituximab-mediated CLL engulfment and also abrogate macrophage spreading and invasive capacity mediated by colony stimulating factor-1 [68, 52].

In line with the T cell paradigm of Th1 and Th2 subsets, macrophages polarize in response to different types of microbial and environmental signals into two subpopulations, i.e., classic inflammatory M1 and alternative immunosuppressive M2 macrophages [69, 70]. The M1 phenotype is characterized by the expression of pro-inflammatory cytokine, high production of reactive nitrogen, stimulation of Th1 response, and potent activity against microbes and tumor cells. Conversely, M2 macrophages show efficient phagocytic activity, high expression of scavenging molecules, mannose and galactose receptors, and involvement in parasite containment, tissue remodeling and tumor promotion. M1 macrophage polarization is mediated by STAT1, the p65 subunit of NF-κB and IRF3, whereas STAT3 and STAT6 mediate M2 macrophage polarization. Btk is a critical signal transducer downstream of TLR4 and interferon γ receptor (IFNGR) by regulating NF-κB and IRF3 activation. As consequence, Btk is involved in macrophage lineage commitment to inflammatory profile. Accordingly, Btk(−/−) macrophages show an impaired ability to polarize into M1 (inflammatory) macrophages through inefficient activation of NF-κB p65 following lipopolysaccharide and IFNγ stimuli, instead showing enhanced induction of M2-associated markers [71]. As an M2 phenotype characterizes tumor-associated macrophages (TAMs) leading to low tumoricidal activity, promotion of angiogenesis and poor prognosis for patients in several tumoral settings [72, 73], it will be of great interest to evaluate the effects of ibrutinib on macrophage polarization. In particular, NLCs are round or fibroblast-shaped adherent cells differentiated from peripheral blood-derived monocytes in vitro and also detected in lymph nodes (LN) of CLL patients [74–76]. NLCs share several features with tumor-associated macrophages and deregulated expression of genes involved in immunocompetence [77, 75, 78, 79]. Lenalidomide was shown to modify the balance of NLCs phenotype from a M2-skewed immunosuppressive towards a M1-skewed inflammatory profile [80]. In this scenario, studies evaluating the effect of ibrutinib on NLCs and the combination with lenalidomide would be useful.

### Osteoclasts

Osteoclasts (OC) originate from bone marrow-derived monocyte/macrophages precursor cells and are strictly regulated by the receptor activator of nuclear factor KB ligand (RANKL), which cooperates with immunoreceptor tyrosine-based activation motif (ITAM) signals to mediate calcium release and then induces the master transcription factor for osteoclastogenesis, the nuclear factor of activated T cells c1 (NFATc1). Btk and Tec are highly expressed in OC and bridge the RANKL and ITAM signals, thus leading osteoclast differentiation [81]. In line with the role of Btk in osteoclast function, studies on Btk mutation disease (XLA in humans and Xid in mice) clearly revealed that Btk-deficent osteoclasts do not completely differentiate into mature multinucleated osteoclasts, leading to defective bone resorption activity [82, 83].

Analogously, the pharmacological blockage of Btk by ibrutinib impairs the RANKL signaling in OC and NFATc1 induction, thus reducing the bone resorption. Noteworthy, ibrutinib treatment significantly inhibits MM-induced osteolysis of implanted human bone chips in SCID mice and ameliorates the bone loss in a RANKL-induced osteoporosis mouse model [32, 84] (Fig. 2). Myeloma cells have a high capacity to induce osteolytic bone lesions in patients, especially in the advance stages, due to the presence of overactive osteoclasts and inactive osteoblasts. In this scenario, ibrutinib may have a potential role in counteract MM-induced osteoclast iperactivation and bone lysis as well as in disruption of mutualistic interaction between MM cells and osteoclasts, by interfering with crucial cytokine and chemokine signals [32, 85]. Ibrutinib generated abnormal giant osteoclasts with defective bone erosion activity and induced OC apoptosis in vitro in long-term culture. Moreover, Btk inhibition by ibrutinib leads to an aberrant organization of actin cytoskeleton critical for bone resorption [32]. It has to be considered that OC obtained from XLA patients showed an impaired bone resorption activity in vitro, but conversely, any evidence of alteration in bone density was detected in patients. This effect seems to be derived from a compensatory mechanism mediated by the up-regulation of several inflammatory cytokines (IL6, IL1β, and TNFα) in XLA patients that re-equilibrates OC function [83]. Ibrutinib was reported to block the secretion of these multiple inflammatory cytokines in MM microenvironment, leading to the potential interruption of the cytokine-mediated compensation of OC activity [32].

## Conclusive remarks

Several novel molecules acting as inhibitors of pivotal tyrosine kinases downstream the BCR signaling cascade and/or involved in B cell trafficking and homing are rapidly spreading in the landscape of therapeutic options in B cell malignancies, in particular in CLL and MCL settings where current standard therapeutic strategies induce substantial toxicity and are not curative, with nearly all patients relapsing. In this scenario, the Btk inhibitor ibrutinib and the PI3Kδ inhibitor idelalisib have demonstrated good safety profile and promising clinical efficacy in different B cell malignancies, clearly affecting the survival of neoplastic B cells by destabilizing the multifactorial platform of microenvironment signals that commonly sustain the malignant clone. These agents also delocalize a consistent fraction of the tumor B cell clone from the protective tissue compartment to the periphery interfering with pathogenetic mechanisms of recirculation and homing. However, it had been immediately clear the limited capacity of these agents to completely eradicate the neoplastic clone, even if an improvement in quality of responses is seen when patients are treated for an extended period. Noteworthy, the molecular targets of ibrutinib and idelalisib are not restricted to B cell compartment but regulate key functions in other cellular elements, i.e., NK, T cells, macrophages and osteoclasts. Neoplastic B cells deeply modify the architecture of infiltrating tissue compartment, skewing the functions of surrounding non-malignant cellular components to generate a protective and nurturing microenvironment. Moreover, they also modify the attitude of cells devoted to natural and adoptive immune response, inducing an immunosuppressive and immune-tolerant behavior. Deepening the effects of TKIs on the normal cellular components in the microenvironment of lymphoproliferative disorders might be of extreme relevance to better understand some unexplained phenomena observed in the clinical setting, first among all, why TKIs are so efficient, able to quickly reduce lymphoid mass, but they can obtain a complete response only in a small minority of cases. Moreover, specific TKI toxicity could find a good explanation.

In conclusion, shedding light into the effects of these TKIs to non-malignant cellular compartment will help to define how they modify the nurturing and protective niches of B cell clone into tissues, giving information for a more efficient and rational use of TKIs and, importantly, suggesting a rational approach for association with other agents as the classical cytotoxic drugs, monoclonal antibodies and immune-modulating agents.

## Abbreviations

ABC: activated B cell-like subtype
ADCC: antibody-dependent cell-mediated cytotoxicity
ADCP: antibody-dependent phagocytosis
BCR: B cell receptor
BR: bendamustine, rituximab
Btk: Bruton tyrosine kinase
CDC: complement-dependent cytotoxicity
CIT: chemoimmunotherapy
CLL: chronic lymphocytic leukemia
CR: complete responses
DLBCL: diffuse large B cell lymphoma
FcR: Fc receptor
FCR: fludarabine, cyclophosphamide and rituximab
FDA: Food and Drug Administration
FL: follicular lymphoma
HCL: hairy cell leukemia
IFN: interferon
IFNGR: interferon γ receptor
IL2: interleukin 2
ITAM: immunoreceptor tyrosine-based activation motif
Itk: inducible T cell kinase
LN: lymph nodes
LPL: lymphoplasmacytic lymphoma
MAPK: mitogen-activated protein kinase
MCL: mantle cell lymphoma
MM: multiple myeloma
MOA: mode of action
MZL: marginal zone lymphoma
NFAT: nuclear factor of activated T cells
NFATc1: nuclear factor of activated T cells c1
NF-κB: nuclear factor κB
NHL: non-Hodgkin lymphoma
NK: natural killer
NLCs: nurse-like cells
OC: osteoclasts
ORR: overall response rate
PFS: progression-free survival
PI3K: phosphoinositol 3 kinase
PLCγ: phospholipase Cγ
PR: partial response
RANKL: receptor activator of nuclear factor KB ligand
R-CHOP: rituximab, cyclophosphamide, doxorubicin, vincristine and prednisone
Rlk: resting lymphocyte kinase
SLL: small lymphocytic lymphoma
Tec: tyrosine kinase expressed in hepatocellular carcinoma
TKIs: tyrosine kinase inhibitors
TLR: toll-like receptor
TNF: tumor necrosis factor
WM: Waldenström macroglobulemia
Xid: X-linked immunodeficiency
XLA: X-linked agammaglobulinemia
